# Supplementary material for: Ghrelin Alleviates Experimental Ulcerative Colitis in Old Mice and Modulates Colonocyte Metabolism via PPARγ Pathway
Source: Int J Mol Sci. 2022 Dec 29;24(1):565. doi: 10.3390/ijms24010565 (PMC9820475; doi:10.3390/ijms24010565)
Supplement: Supplementary file 1 [file ijms-24-00565-s001.zip › ijms-2114463-supplementary.pdf]

# Supplement figure

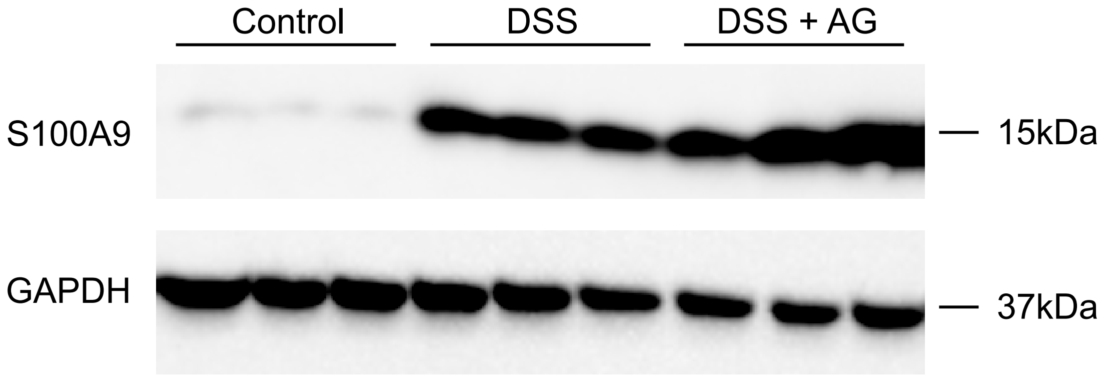


**Figure S1**. Ghrelin treatment did not affect colon inflammation as assessed by S100A9 expression in the early recovery phase. Colon homogenates from mice in DSS or DSS+AG groups collected on day 8 as well as control group were assayed using western blot analysis.
